# Characteristics and outcome of cardiopulmonary resuscitation in hospitalised African children^☆^

**DOI:** 10.1016/j.resuscitation.2008.09.019

**Published:** 2009-01

**Authors:** A. Olotu, M. Ndiritu, M. Ismael, S. Mohammed, S. Mithwani, K. Maitland, C.R.J.C. Newton

**Affiliations:** KEMRI/Welcome Trust Collaborative Research, Centre for Geographic Medicine Research, Coast, Kilifi, Kenya

**Keywords:** Cardiopulmonary arrest, Child, Resuscitation, outcome

## Abstract

**Objective:**

To review the characteristics and outcome of cardiopulmonary resuscitation in children at a rural hospital in Kenya.

**Patients and method:**

All children aged 0–14 years who experienced ≥1 episode of respiratory or cardiopulmonary arrest during April 2002–2004 were prospectively identified. Demographic variables, cause of hospitalisation, type and duration of arrest, resuscitation measures taken and outcomes were determined.

**Results:**

114 children experienced at least one episode of respiratory arrest (RA) or cardiopulmonary arrest (CPA). Cardiopulmonary resuscitation (CPR) was performed on all children. “Do not resuscitate order” (DNR) was given in 15 patients after initial resuscitation. Eighty two patients (72%) had RA and 32 (28%) had CPA. 25/82 (30%) patients with RA survived initial CPR compared to 5/32 (16%) with CPA. Survival at discharge was 22% (18/82) in children who had RA while no one with CPA survived at discharge. The leading underlying diseases were severe malaria, septicaemia and severe malnutrition. Prolonged resuscitation beyond 15 min and receiving adrenaline [epinephrine] (at least one dose of 10 μg/kg IV) were predictive of poor final outcome.

**Conclusion:**

Cardiopulmonary arrest after admission has a very poor prognosis in our hospital. Infectious diseases are the main underlying causes of arrest. If a child fails to respond to the basic tenements of PALS within 15 min then it is unlikely that further efforts to sustain life will be fruitful in hospitals where ventilation facilities are not present.

## 1 Introduction

Paediatric cardiopulmonary resuscitation in African rural hospitals presents a great challenge to health workers who are faced with inadequate facilities and often advanced pathophysiology in sick children. The outcome of children after cardiac or respiratory arrest is better than that of adults, but in resource poor countries that outcome is very poor.^1,2^

Deficiency in arrest management is one of the factors which could influence the outcome of resuscitation. In addition to knowledge on paediatric resuscitation, survival from resuscitation depends on several other factors including underlying disease, the time elapsing between arrest and the initiation of resuscitation, and the duration of cardiopulmonary resuscitation.^3–6^

There has been an increase in the number of clinicians and nurses receiving training in Paediatric Advanced Life Support both as an in-house course and in other centres of paediatric excellence in our setup. Coupled with the training was the availability of more Bag Valve Masks, intubation sets and the regular supply of resuscitation equipment and drugs. We therefore set up a prospective study to determine the outcome and risk factors for an unsuccessful resuscitation in this hospital.

## 2 Patients and methods

### 2.1 Study settings

Kilifi District Hospital is a 60-bed secondary care hospital serving the population of approximately 200,000 people in Kilifi District, Kenya. It has a seven-bed Paediatric High Dependency Unit (PHDU), which admits all critically ill children under 13 years of age.

### 2.2 Resuscitation procedure

All arrests in the PHDU are managed by the attending clinician and nurses. There is no anaesthetist in the hospital. The PHDU has an emergency cart that contains the necessary equipment and medications for Paediatric Advance Life Support (PALS) but there are no defibrillators or facilities for ventilation. The nursing staffs that initiate resuscitation are trained in basic life support while the clinicians and paediatricians are responsible for advanced life support procedures. Most of the resuscitation is monitored on Siemens SC 7000 cardiac monitors (Siemens, UK). The resuscitation protocol in the hospital is adapted from the Paediatric Advanced Life Support of the Resuscitation Council UK.

### 2.3 Types of arrest

The types of arrest were characterised into: respiratory arrest (RA) defined as the absence of spontaneous ventilation in a presence of palpable pulses in major arteries, cardiopulmonary arrest (CPA) defined as documented absence of spontaneous ventilation and palpable pulses in major arteries.

### 2.4 Demographic and clinical information

All demographic and clinical data are held in an integrated database and in hard copy records. Clinicians keep daily progress notes on patients and events surrounding arrests. Beginning January 2004, information on any arrest and resuscitation was also immediately transcribed onto a proforma. Data was prospectively collected and included the time and type of arrest, whether any resuscitation measures were undertaken; the type of resuscitation given, time of commencement, duration and drugs administered during the resuscitation. The outcome of the resuscitation was recorded immediately following the resuscitation and survival to the time of discharge from the hospital was documented.

### 2.5 Data analysis

Data were entered and analysed using Epi info (CDC, GA) program version 3.2.2. The proportion of children having respiratory and cardiopulmonary arrest and the outcome in these groups was compared by the Chi-squared test (*χ*^2^), *χ*^2^-test for trend and Fisher's exact test. We also performed a multivariate analysis of the survival immediately after resuscitation or to the time of discharge. We included sex, age, type of arrest, presence of bacteraemia and number of arrests. Premature children or those who had no resuscitation were excluded from multivariate analysis. Results were judged as reaching statistical significance at *P*-value ≤0.05.

## 3 Results

A total of 114 patients arrested at least once, with 15 (13%) suffering from more than one arrest. The median age was 28 (0–144) months at admission and 68 (60%) were males (Table 1). The “do not resuscitate” order was given in 15 children (13%) of whom all were post arrest orders. All of the children with “do not resuscitate” orders were suffering from severe malnutrition and were in their terminal stages. Out of the 114 children, 30 (26%) survived after initial attempt. At discharge, only 18 (16%) were alive.

There were no differences in CPR outcome within the years. There was no difference between PALS trained and BSL-only trained clinicians/nurses in terms of final outcome of CPR.

Laboratory investigations 12 h before the initial arrest showed no difference in electrolyte and metabolic status by final survival status (Table 2). However, children with malnutrition had significant lower potassium levels compared to other children (mean 3.3 versus 4.5 mmol/l, *P*-value = 0.003).

### 3.1 Type of arrest and outcome

Eighty two (72%) patients had respiratory arrest and 32 (28%) had cardiopulmonary arrest. Among neonates there were 16/20 (80%) and 4/20 (20%) cases of RA and CPA respectively. The main underlying conditions in neonates with arrest were septicaemia 10/20 (50%), and neonatal tetanus 5/20 (25%). Premature neonates and children with resuscitation of birth events were not included in the analysis.

Following resuscitation attempts, initial survival occurred in 25/82 (30%) after respiratory arrest, compared to 5/32 (16%) cases after CPA (*P* = 0.2). At discharge, 18 children (22%) with respiratory arrest survived while none of the children with CPA survived (*P* = 0.003) [Table 3]. All children with RA who died had subsequent cardiopulmonary arrest. One patient with respiratory arrest had weakness of the right lower limb at discharge. The underlying disease in this patient was severe malaria.

### 3.2 Characteristics of resuscitation

The median time of resuscitation was 36 min (5–180). There was no difference in mean duration of resuscitation by type of arrest. Prolonged resuscitation of more than 15 min was associated with poor outcome during CPR, both immediate (*P*-value = 0.04) and at discharge (*P*-value = 0.001).

Adrenaline [epinephrine] (at least one dose of 10 μg/kg IV) was administered in 39/114 (34%) patients. Survival rate at discharge in a group of children who received adrenaline was significantly lower [Table 3].

Intubations were attempted in 32 patients who had insufficient ventilation by bag and mask technique. The success rate was 86% (28/32). The intubation was followed by assisted ventilation. The median duration of assisted ventilation was 33 min (10–180). Survival among intubated children was 25% (7/28) compared to 17.2% (15/87) in those with no or failed intubation attempts (Table 3).

### 3.3 Underlying diseases

Severe malaria, septicaemia and severe malnutrition were generally the most common underlying diseases (Table 4). The leading causes of arrest and/or death among neonates were septicaemia and neonatal tetanus while in infants severe pneumonia was the most common diagnosis followed by septicaemia. Severe malaria was common in children above 1 year of age.

Multivariate analysis of the potential factors associated with poor survival revealed that longer duration of resuscitation of more than 15 min and receiving adrenaline (at least one dose of 10 μg/kg IV) were independent predictors of poor outcome at discharge. The ratio (95% confidence interval) for final survival was 0.02 (0.002–0.3) and 0.3 (0.001–0.6) respectively.

## 4 Discussion

This was the first prospective study looking at characteristics and outcome of cardiopulmonary arrest in Kilifi District Hospital. A total of 114 patients arrested at least once and had attempted resuscitation. The most common type of arrest was respiratory in nature. Initial outcome of CPR was poor for both types of arrests with only 30% and 16% of children surviving for respiratory and cardiopulmonary arrest, respectively. Survival to discharge only occurred in children with respiratory arrest. Prolonged duration of CPR of more than 15 min and receiving adrenaline (at least one dose of 10 μg/kg IV) appear to be independent predictors of poor outcome. Metabolic and electrolyte status 12 h before initial arrest were similar in all children suggesting the advanced disease at admission is not the only factor influencing the outcome of CPR. The most common underlying diseases were severe malaria, septicaemia and severe malnutrition.

### 4.1 Outcome of arrest

Respiratory arrest remains the main type of arrest in our children. Final survival in patients with respiratory arrest has remained poor. A previous study in Kilifi had shown a survival rate of 15% among patients with respiratory arrest. Although this study has shown an improved survival rate of 22% in patients with respiratory arrest, overall survival from resuscitation in our children remains poor compared to developed countries.^7,5,6,8^

Advanced pathophysiology was described as one of the reasons for the poor outcome; however, we found all children to have similar metabolic and electrolyte status 12 h before initial arrest regardless of their final outcome. Analysis of laboratory data 5–10 h before arrest showed no difference in electrolyte and metabolic status by final outcome at discharge either (data not shown). This suggests advanced pathophysiology alone could not have contributed to the low survival. Factors related to the management of resuscitation, post-resuscitation management and treatment of underlying diseases constitute a more plausible explanation.

Neurological sequela was documented in only one patient who had severe cerebral malaria and suffered a respiratory arrest. However, this should be viewed with caution as lack of inadequate neurological examination and documentation might have missed some cases of mild neurological sequelae.

An effort to train nurses and clinicians on Paediatric Advance Life Support may have resulted in improved knowledge on the resuscitation. The paediatric advanced life support course significantly increases immediate short-term knowledge of paediatric resuscitation for all professional groups^9^; however, recertification is important to ensure the concepts of PALS are retained.

### 4.2 Underlying diseases

Infectious diseases were the most common underlying diagnoses in our children. In general, infectious diseases are the main cause of admission and death in young infants^10^ and this is reflected in the aetiologies underlying the arrests. Prompt and appropriate treatment of diseases is important to avoid complications. Prevention and management of the most common infectious diseases remains the main challenge in developing countries^11^ and its success would reduce associated complications which usually culminate in arrest. Severe malaria was the leading underlying disease. New interventions in managing children with severe malaria, including effective fluid interventions, could prevent complications which usually culminate in arrest.^12^

### 4.3 Characteristic of resuscitation

The duration of resuscitation was negatively correlated with final survival which is consistent with other studies.^13,6^ Although cessation of resuscitation should not only depend on the duration of resuscitation, our results support findings in other studies that effective resuscitation without spontaneous return of circulation for more than 15–25 min indicates a grave prognosis.^14^ Duration of resuscitation should also be guided by the extent of underlying disease and pre-existing organ failure. Prolonged resuscitation is associated with reduced blood flow and in most cases can result in neurological impairment.^15^ Therefore, the nature and duration of any resuscitation should consider the impact of resuscitation on long-term neurological status.

The outcome of patients who received adrenaline was poor compared to those who did not receive it. Similar findings have been reported in resuscitation outcomes for patients admitted in a paediatric intensive care unit.^6^ This was evident in children who had delayed arrest although there was no difference in terms of final survival in those who had suffered immediate arrest. However, adrenaline injection tends to be given in severely ill children which could explain the trend.

Intubation attempts were carried out in only 32/135 (23.7%) patients who were resuscitated. Survival in children who were intubated showed a trend towards better outcome as compared to children who were not intubated. Intubation is a recognised way of securing an airway and if effectively performed, and combined with bag-mask ventilation, may improve outcome in children who arrest. However, long-term ventilation requires ventilators, something bag-mask ventilation cannot achieve.

### 4.4 Limitations

Due to lack of follow up information we could not ascertain the long-term outcome of patients who survived at discharge.

## 5 Conclusion

Cardiopulmonary arrest after admission has a very poor prognosis in our hospital. Efforts to improve this will be unlikely to change the prognosis in this group unless this can be supported by the broader provision of ventilation facilities and management directed towards early identification and prospective intubation and ventilation for cases where effect management is constrained by safety in an unventilated child e.g. seizure control, more aggressive volume expansion and inotropic support. However, this is unlikely to be widespread in district government hospitals without significant economic improvements. In the meantime, efforts to resuscitate on admission (the ‘Golden Hour’) are likely to make a significant impact on outcome and should be implemented more widely. Resuscitation for CPA after admission is unlikely to achieve much benefit in child survival since our data suggest that mortality is almost universal. Nevertheless, attempts should be made. If the child fails to respond to the basic tenements of PALS then it is unlikely that further efforts to sustain life will be fruitful in hospitals where ventilation facilities are not present.

## Conflict of interest statement

None.

## Figures and Tables

**Table 1:** Characteristics of the study population and their relationship to survival at discharge.

	Total number of patients (*n* [%])	Survival at discharge/total at each category (*n* [%])	Chi-squared test	*P*-value
Age				
<1 year	52(46)	10/52(19)	0.9	0.7
1–5 years	44(37)	6/44(14)		
5–14 years	18(16)	2/18(11)		

Sex				
Female	46(40)	6/46(13)	0.4	0.6
Male	68(60)	12/68(18)		

Initial site of admission				
General ward	26(23)	2/26(8)	1.6	0.2
HDU^a^	88(77)	16/88(18)		

Blood transfusion				
Yes	34(30)	5/34(15)	0.05	1
No	69(70)	13/69(16)		

a High Dependency Unit.

**Table 2:** Electrolyte and metabolic status 12 h prior to arrest.

Outcome at discharge				
	Total	Alive	Died	*P*-value*
Base excess	30	−13(−18,−7)[8]	−12(−16,−7)[22]	0.7
pH	30	7.1(6.9–7.2)[8]	7.2(7.1–7.3)[22]	0.3
Potassium	35	5(3.6–6.4)[9]	4.4(3.8–5)[26]	0.3
Sodium	35	134(128–140)[9]	133(129–136)[26]	0.6
pCO_2_	30	5.6(3.5–7.6)[8]	4.2(3–5.2)[22]	0.2

All laboratory tests performed 12 h before arrest. [] Indicate number of patients in each group.

* *P*-value calculated from the Chi-square value for the two groups.

**Table 3:** Characteristics of the CPR event and the relationship to survival at discharge.

	Total number of patients (*n* [%])	Survival at discharge/total at each category (*n* [%])	Chi-squared test	*P*-value
Arrest type				
Respiratory	82(72)	18/82(22)	8.3	0.003
Cardiopulmonary	32(28)	0/28(0)		

Duration of resuscitation				
0–15 min	13(11)	7/13(54)	16	0.001
>15 min	101(89)	11/101(11)		

Adrenaline^a^				
Yes	39(34)	1/39(3)	7.8	0.005
No	75(66)	17/75(23)		

Intubations				
Yes	27(24)	7/27(25)	0.8	0.2
No	87(76)	15/87(17.2)		

Septicaemia^b^				
Yes	9(8)	1/9(11)	0.2	1
No	105(91)	17/105(16)		

Underlined *P*-values are significant.

a At least one dose of 10 μg/kg IV.

b Based on conformed blood culture results.

**Table 4:** Underlying diseases during CPR.

Diagnosis^a^	Number	Percentage
Severe malaria	20	17.5
Septicaemia	16	13.2
Severe malnutrition	14	12.3
Encephalopathy—unknown cause	10	8.8
Meningitis—not TBM	9	7.9
Severe anaemia	9	7.9
Severe pneumonia	8	7
Gastroenteritis	6	5.3
Neonatal tetanus	5	4.4
Other causes	18	16.8

TBM: Tuberculous meningitis.

a Based on final diagnosis at discharge/death.
