# Supplementary material for: Characteristics and outcome of cardiopulmonary resuscitation in hospitalised African children
Source: Resuscitation. 2009 Jan;80(1-3):69–72. doi: 10.1016/j.resuscitation.2008.09.019 (PMC2706393; doi:10.1016/j.resuscitation.2008.09.019)
Supplement: Supplementary file 1 [file mmc1.doc]

Resumen

*Objetivo*: Revisar las características y resultado de la reanimación cardiopulmonar (RCP) en niños en un hospital rural de Kenia.

*Pacientes y métodos*: Se identificaron prospectivamente todos los niños en edades de 0-14 que experimentaron ≥ 1 episodio de paro respiratorio o cardiaco durante Abril 2002-2004.Se determinaron variables demográficas, causas de hospitalización, tipo y duración del paro, medidas de resucitación tomadas y resultados.

*Resultados*: Un total de 114 niños experimentaron al menos un episodio de paro respiratorio (RA) o paro cardiorrespiratorio (CPA). En todos los niños se realizó reanimación cardiopulmonar (RCP). Se dio “orden de no resucitar”(DNR) en 15 pacientes después de la reanimación inicial. Ochenta y dos pacientes (72%) tenían RA y 32 (28%) tenían CPA. Sobrevivieron la reanimación inicial 25/82 (30%) de los pacientes con RA comparados con 5/32 (16%) con CPA> La sobrevida al alta fue 22% (18/82) en niños con RA, mientras ninguno con CPA sobrevivió al alta. La principales enfermedades subyacentes fueron malaria severa, septicemia y desnutrición severa. Una reanimación prolongada más allá de 15 minutos y el recibir adrenalina [epinefrina] (al menos una dosis de 10 μg/kg IV) fueron predictivas de resultado final pobre.

*Conclusión*: El paro cardiorrespiratorio después de la admisión tiene muy mal pronóstico en nuestro hospital.Las enfermedades infecciosas son la principales causas de paro. Si un niño no responde a las maniobras básicas de PALS dentro de 15 minutos entonces es improbable que ulteriores esfuerzos para mantenerlo con vida sean fructíferos en hospitales donde no hay facilidades de ventilación.

© 2008 Publicado por Elsevier Ireland Ltd.

*Palabras clave*: Paro Cardiaco intrahospitalario; Pediatría; Reanimación cardiopulmonar (RCP); Hospital Rural; Resultado; Kenia
